# Production and Immune Response Against Pandemic Influenza Candidate Vaccines as Preparedness Against the Circulating H5N1 Influenza Viruses

**DOI:** 10.3390/vaccines13060620

**Published:** 2025-06-08

**Authors:** Paulo Lee Ho, Yordanka Medina-Armenteros, Lívia Mendonça Munhoz Dati, Daniela Cajado-Carvalho, Christian Savio Silva, Pollyanna Fernandes Campos, Patrícia Antonia Estima Abreu, Júlia Tavares de Castro, Paulo Newton Tonolli, Mahyumi Fujimori, Rhubia Silveira Martins Rosa, Soledad Palameta, Michael Edward Miller, Vitor Anselmo Sakihara, Fernanda de Lima Valadares, Fabiana Lauretti Ferreira, Bianca Pereira Carvalho Holanda, Douglas Gonçalves de Macedo, Priscila Comone, Natully de Souza Suffert Fogaça, Alexandre Bimbo, Felipe Catanzaro De Moraes, Stephane Tereza Queiroz de Andrade, Helena Lage Ferreira, Edison Luiz Durigon, Clarice Weis Arns, Esper George Kallás, Milena Apetito Akamatsu, Ricardo das Neves Oliveira

**Affiliations:** 1 BioIndustrial Center, Butantan Institute and Butantan Foundation, São Paulo 05503-900, SP, Brazil; yordanka.armenteros@fundacaobutantan.org.br (Y.M.-A.); livia.dati@fundacaobutantan.org.br (L.M.M.D.); daniela.carvalho@fundacaobutantan.org.br (D.C.-C.); christian.silva@fundacaobutantan.org.br (C.S.S.); pollyanna.campos@fundacaobutantan.org.br (P.F.C.); patricia.aniz@butantan.gov.br (P.A.E.A.); julia.castro@fundacaobutantan.org.br (J.T.d.C.); paulo.tonolli@fundacaobutantan.org.br (P.N.T.); mahyumi.fujimori@fundacaobutantan.org.br (M.F.); vitor.sakihara@fundacaobutantan.org.br (V.A.S.); fernanda.valadares@fundacaobutantan.org.br (F.d.L.V.); fabiana.ferreira@fundacaobutantan.org.br (F.L.F.); bianca.carvalho@fundacaobutantan.org.br (B.P.C.H.); douglas.macedo@fundacaobutantan.org.br (D.G.d.M.); priscila.comone@fundacaobutantan.org.br (P.C.); natully.fogaca@fundacaobutantan.org.br (N.d.S.S.F.); alexandre.bimbo@fundacaobutantan.org.br (A.B.); felipe.moraes@fundacaobutantan.org.br (F.C.D.M.); stephane.andrade@fundacaobutantan.org.br (S.T.Q.d.A.); esper.kallas@butantan.gov.br (E.G.K.); ricardo.oliveira@fundacaobutantan.org.br (R.d.N.O.); 2 Institute of Biology, University of Campinas, Campinas 13083-862, SP, Brazil; rhubiasilveiramartins@gmail.com (R.S.M.R.); palametasoledad@gmail.com (S.P.); mikevetbr@gmail.com (M.E.M.); arns@unicamp.br (C.W.A.); 3 Faculdade de Zootecnia e Engenharia de Alimentos, Universidade de São Paulo, Pirassununga 13635-900, SP, Brazil; hlage@usp.br; 4 Instituto de Ciências Biomédicas, Universidade de São Paulo, São Paulo 05508-000, SP, Brazil; eldurigo@usp.br

**Keywords:** influenza virus, H5N1 pandemic influenza vaccine, squalene-based emulsion adjuvant, immunogenicity, hemagglutination inhibition, microneutralization

## Abstract

**Background/Objectives:**H5N1 influenza viruses are spreading worldwide and threaten global public health. Preparedness is necessary to mitigate the worst-case scenario should an H5N1 influenza pandemic occur and justify the development of vaccines against circulating H5N1 viruses of concern. Methods: The production and characterization of egg-based split and inactivated H5Nx of three distinct monovalent antigens from clades 2.3.4.4b, 2.3.2.1c, and 2.3.4 were performed at an industrial scale. These antigens were formulated and their immune responses, when combined or not with IB160 squalene-based oil-in-water emulsion adjuvant in a rat model, were evaluated in a one- or two-dose immunization schedule. IgG antibodies, hemagglutination inhibitions, and microneutralization titers were measured for vaccine-induced immunity and cross-reactivity. Results: Three monovalent vaccines from clades 2.3.4.4b, 2.3.2.1c, and 2.3.4 were produced at an industrial scale and characterized. The immune responses against the monovalent vaccines showed a clade-specific antibody response and the need to combine with IB160 adjuvant for a required immune response. Conclusions: Considering the candidate vaccine viruses (CVVs) with the testing potency reagents available and that the antibody response obtained against the CVVs produced was clade-specific, IDCDC RG-71A is the indicated CVV for the predominant currently circulating H5N1 influenza virus of clade 2.3.4.4b and must be combined with adjuvant to induce a higher and efficacious immune response in a two-dose immunization protocol.

## 1. Introduction

Since the end of 2021, Highly Pathogenic Avian Influenza (HPAI) H5N1 viruses of clade 2.3.4.4b have been circulating in Europe, Asia, North America, South America, Africa, and remote regions like Antarctica [1]. This virus has infected birds (wild and domestic) and spilled over to non-human mammals [2], with mass death events reported in minks [3], harbor seals [4], and sea lions [5]. Reports of H5N1 infections in cats in Poland [6], South Korea [7], and the United States (USA) [8] are also of particular concern. This domestic animal lives in close contact with people, frequently both indoors and outdoors, increasing the risk of transmission from non-human mammals to humans. The likelihood of the virus acquiring the capacity to infect and transmit among humans reinforces its pandemic potential, since the population is naïve to the H5 antigen. Human infections were reported in the USA due to contact with infected dairy cows. Indeed, the infected cows could transmit the virus through the milk. Up to 21 April 2025, the USA reported 70 human cases; 41 had direct contact with the infected cattle, 24 with poultry, two with other animal exposure, and three with an unknown animal source [9]. Most of the cases presented mild symptoms. However, illness severity can range from mild to critical illness, and even to death [9,10]. Furthermore, although human zoonotic infections with H5N1 have been reported as sporadic cases associated with contact with infected animals, the virus has a high dispersion capacity, infects a very diverse number of different types of vertebrates, and has reached nearly global distribution, making HPAI H5N1 clade 2.3.4.4b a dangerous threat [11].

The H5N1 virus currently circulating is derived from A/goose/Guangdong/1/96 (GS/GD) lineage [12]. The evolution of GS/GD viruses diversified the hemagglutinin (HA) antigen and, based on H5 sequences, the viruses are classified into genetic groups (clades and subclades) [13]. The nature of the influenza genome—segmented RNA—allows it to give rise to these variants (clades and genotypes) and/or subtypes by means of minor (drift) and major (shift) changes, respectively. Minor changes occur in the whole genome but are more evident in genes encoding HA and neuraminidase (NA). The combination of these proteins determines the subtype and its antigenic characteristics [14]. Antigenic drift is generally associated with seasonal influenza, and antigenic shift with pandemic influenza. When more than one virus genotype infects a single host cell, the associated influenza genes can reassort in co-infected cells (shift), resulting in the emergence of antigenically novel viral strains and/or subtypes to which the human population is naïve [14]. As with other influenza vaccines, a vaccine for HPAI H5N1 virus is expected to be immunogenic and able to induce antibodies against these major antigenic determinants.

**Figure 1:**
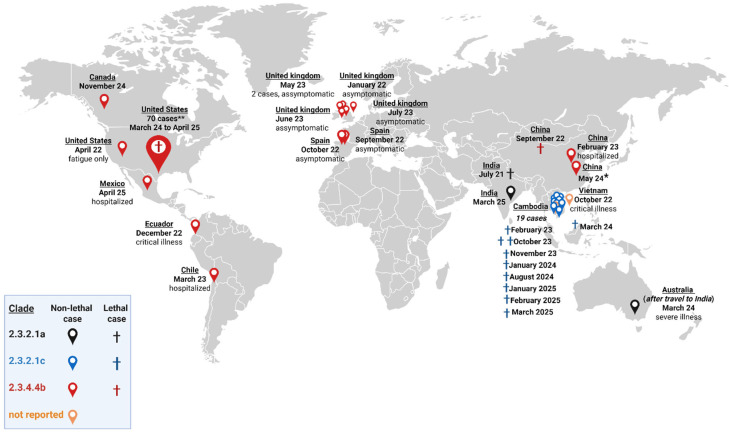
Geographical distribution of human HPAI H5N1 infections by clade from July 2021 to 21 April 2025. Total cases (N = 109). Clade 2.3.2.1a (black; N = 3): EPI_ISL_14223821, India, July 2021, 11-year-old male, critical illness, death 12 July 2021; EPI_ISL_19156871, Australia, after travel to India, 6 March 2024, severe illness, 2-year-old female; and EPI_ISL_19836227, India, 7 March 2025, 2-year-old female, death. Clade 2.3.2.1c (blue; N = 19): EPI_ISL_17024123, Cambodia, 21 February 2023, 11-year-old female, death; EPI_ISL_17069010, Cambodia, 23 February 2023, 49-year-old male, mild illness; EPI_ISL_18373263, Cambodia, 6 October 2023, 2-year-old female, death; EPI_ISL_18366401, Cambodia, 7 October 2023, 54-year-old male, death; EPI_ISL_18540514, Cambodia, 23 November 2023, 21-year-old female, death; EPI_ISL_18543642: Cambodia, 24 November 2023, 5-year-old female; EPI_ISL_18823967, Cambodia, 23 January 2024, 3-year-old male; isolate ID not published, Cambodia, 28 January 2024, 69-year-old male; EPI_ISL_18879683, Cambodia, late January, 9-year-old male, death; EPI_ISL_19312044, Cambodia, 8 February 2024, 16-year-old male; EPI_ISL_19031556: Vietnam, 19 March 2024, 21-year-old male, death; and two cases with isolate ID not published in Cambodia (Takeo province), a 3-year-old boy and a 5-year-old girl; EPI_ISL_19312043, Cambodia, 30 July 2024, 4-year male; EPI_ISL_19312044, Cambodia, 2 August 2024, 16-year female; EPI_ISL_19353003, Cambodia, 17 August 2024, 15-year female, death; EPI_ISL_19661054, Cambodia, 8 January 2025, 28-year-old man, death; EPI_ISL_19752030, Cambodia, 24 February 2025, 2-year-old male, death; and EPI_ISL_19791427, Cambodia, 22 March 2025, 3-year-old male, death. Clade 2.3.4.4b (red; N = 85): EPI_ISL_8799552, United Kingdom (UK), 26 Dezember 2021, 79 years old, asymptomatic; ON759331.1, USA (Colorado), 20 April 2022, fatigue only; EPI_ISL_15542438, Spain, 23 September 2022, 19-year-old male, asymptomatic; isolate ID not published (identified by association with poultry outbreaks), China, September 2022, death; EPI_ISL_16813290, Spain, 13 October 2022, 27-year-old male, asymptomatic; EPI_ISL_17021605, Ecuador, 5 January 2023, critical illness; EPI_ISL_17075747, China, 31 January 2023, 53-year-old woman, hospitalized, survived; EPI_ISL_17468386, Chile, 24 March 2023, 53-year-old male, critical illness; EPI_ISL_17736680, UK, 4 May 2023 ; EPI_ISL_17736649, UK, 6 May 2023; EPI_ISL_17980947, UK, 21 June 2023; EPI_ISL_18161874, UK, 7 July 2023; isolate from China, May 2024 (* ID isolate and clinical data not available); EPI_ISL_19548836, Canada, 9 November 2024, 13-year-old girl; EPI_ISL_19695821, UK, January 2025; and isolate from Mexico, 18 March 2025, 3-year-old female, death (isolate ID not published). ** From March 2024 to April 2025 additional A (H5N1) cases have been identified in persons exposed to dairy cows and poultry in the USA. Seventy isolates were reported in ten states, with one death in Louisiana. Not reported clade (orange; N = 2): Vietnam, October 2022, critical illness and Cambodia, 21 February 2024, 17-year-old female (both isolate IDs not published) [9,11,18,19,20,21,22]. The figure was created with Biorender.com.

Nowadays, there are human-licensed H5N1 vaccines based on candidate vaccine viruses (CVVs) belonging to clades 1 (NIBRG-14 and SJRG-161052), 2.1.3.2 (IBCDC-RG2), and 2.2.1 (NIBRG-23) [15,16]. Nevertheless, these vaccines’ antigenic prototypes differ from the prevalent H5N1 virus related to H5 clade 2.3.4.4.b. The virus has been detected in various bird and mammal species and on every continent, except Australia and New Zealand [1]. It is known that different clades do not induce cross-neutralization; hence, it is conceivable that the already-registered vaccines will not protect against current H5N1 infection. [17] Thus, pre-pandemic H5N1 vaccines must target, at first sight, the predominant circulating clades (the case of clade 2.3.4.4b), as well as those causing a high percentage of fatal human cases (Figure 1) [9,11,18,19,20,21,22]. This is the case of clade 2.3.2.1c with a more restricted circulation, identified in Southeast Asia, responsible for ten of the twelve fatal human cases related to H5N1 viruses in the ongoing epizootic [11,20]. Comparing the H5 amino acid sequences of the recent H5N1 bird isolates and human cases with those of the CVVs [23], we have selected three CVVs for the development of H5 vaccines as preparedness against a potential H5N1 pandemic [24,25]. They are the best CVVs matching H5 clades 2.3.4.4b and 2.3.2.1c, IDCDC-RG71A (A/Astrakhan/3212/2020 (H5N8)) and NIBRG-301 (A/duck/Vietnam/NCVD-1584/2012 (H5N1)), respectively, and a third CVV belonging to clade 2.3.4, IBCDC-RG6 A/Anhui/1/2005 (H5N1), a basal but more distant clade from these circulating viruses. In the present study, we describe the production, characterization, and induced immune response of these three produced monovalent antigens formulated or not with IB160 squalene oil-in-water emulsion adjuvant [26] as a step for an H5N1 vaccine development for pandemic preparedness.

## 2. Materials and Methods

### 2.1. Sequence Analysis

The HA amino acid sequences of H5 were accessed at the GISAID EpiFlu [20] or GenBank [21] databases. The sequences were analyzed using the tools Symple Phylogeny at EMBL services [27] and Protein BLAST (+ 2.15.0 version) at the National Center for Biotechnology Information (NCBI) [28].

### 2.2. Strains

Candidate vaccine viruses A/Anhui/1/2005 (H5N1) [IBCDC-RG6] (clade 2.3.4) and A/Astrakhan/3212/2020 (H5N8) [IDCDC-RG71A] (clade 2.3.4.4b) were obtained from the Centers for Disease Control and Prevention (CDC, Atlanta, GA, USA), and the CVV A/duck/Vietnam/NCVD-1584/2012 (H5N1) [NIBRG-301] (clade 2.3.2.1c) was obtained from the UK National Institute of Biological Standards and Control (NIBSC, Potters Bar, UK) [29,30]. All of them were produced in a PR8 influenza strain backbone by reverse genetics [20,21].

### 2.3. Vaccine Preparation

The large-scale industrial production of the split and inactivated H5Nx antigens was performed under current Good Manufacturing Practices (cGMP) conditions. Briefly, the diluted H5Nx CVV strains from working seed banks were inoculated in 346,752 controlled embryonated eggs (9–11 days) and incubated at 33–35 °C for 60–72 h. After incubation, the eggs were chilled overnight at 2–8 °C, and the allantoic liquid (AL) was harvested, clarified by continuous centrifugation, concentrated, and diafiltrated (cut off 300 KDa). The concentrated virions were purified by two consecutive industrial ultracentrifugation steps of sucrose gradient, and the fractions of interest were collected, diluted with phosphate buffer saline (PBS), and fragmented using Triton X-100 (Merck, Darmstadt, Germany) (0.5% final concentration). The split H5Nx virions were further clarified by continuous centrifugation and diafiltration (cut off 50 kDa, using 10 times the initial volume of PBS). After appropriate dilution, formaldehyde was added to a 0.01% final concentration for inactivation. The H5Nx antigens were filtered in a 0.22 μm filter and considered the concentrated H5Nx vaccine antigen bulk (drug substance). Quality control of H5Nx vaccine antigens was performed according to the WHO guidelines [31,32].

### 2.4. Determination of HA Content

Single Radial Immunodiffusion technique was performed following the protocol of Schild and colleagues [33]. The average concentration of HA expressed as µg/mL, the number of doses per lot, and the number of doses per egg of three lots produced from each CVV strain were calculated.

### 2.5. Vaccine Immunization

Experimental protocols were approved by the Committee on Ethics in Animal Experimentation of Butantan Institute (CEUAIB), protocol number 8665030323, which includes the three CVV strains analyzed. The study was performed according to the guidelines outlined by the Brazilian National Council for Control of Animal Experimentation (CONCEA). Male rats (Wistar strain) aged three months were purchased from ANILAB Veterinarian Laboratory (Paulínea, SP, Brazil). The antigen was administered once or twice, with different doses ranging from 7.5–90 µg/dose, and in the presence or absence of the IB160 adjuvant [26], resulting in 14 immunization conditions for each CVV and a total of 42 experimental groups (Table 1). A vaccine formulation volume of 0.5 mL was administered intramuscularly into the quadriceps muscle of the animal’s hind paw with the formulations containing 7.5, 15, or 60 µg/dose. In the 90 µg/dose, the final volume was 0.75 mL, divided into two inoculations in the right and left paw (0.375 mL/muscle). The vaccine formulation with IB160 oil-in-water emulsion adjuvant was performed by adding an equal volume of the vaccine antigen (in 0.25 mL) to the volume of IB160 adjuvant (0.25 mL) to a final formulation volume of 0.5 mL. The second immunization was performed 21 days apart. Twenty-eight days after the last immunization, the animals were anesthetized with 90 mg/kg of ketamine and 10 mg/kg of xylazine, both from Syntec, Barueri, SP, Brazil. The exsanguination was performed via cardiac puncture and the blood was collected and incubated at 37 °C for 30 min after a conditioned procedure at 4 °C for 2 h for serum collection. Samples were centrifuged at 2000 rpm, 4 °C for 10 min, and the sera were collected and stored at −80 °C for posterior analysis.

### 2.6. Enzyme-Linked Immunosorbent Assay (ELISA)

IgG antibodies against H5Nx antigens were titrated in sera of immunized animals using the ELISA method. The 96-well plates (Nunc MaxiSorp™ cat# 439454, ThermoFisher Scientific,Rochester, New York (NY), USA) were incubated with 1 µg of H5Nx antigens diluted in PBS, pH 7.4 at 4 °C for 18 h. Blocking the unspecific reactive sites was performed with 10% BSA in PBS-T (PBS containing 0.05% Tween 20) at 37 °C for 2 h. Plates were washed three times with PBS-T and incubated with serial dilutions of the pooled sera in 1% PBS-T BSA for 1 h 30 min at 37 °C. After three additional washes with PBS-T, anti-rat IgG (Sigma-Aldrich, St Louis, MO, USA, Cat# A9037, RRID:AB_258429) conjugated to horseradish peroxidase (HRP) was added, and plates were incubated for 1 h at 37 °C. Plates were washed three times with PBS-T, and reactions were developed using o-phenylenediamine dihydrochloride (Sigma, St Louis, MO, USA) as substrate in citrate phosphate buffer, pH 5.0. Reactions were stopped by adding 50 µL of 2.0 M H_2_SO_4_ (Merck, Darmstadt, Germany). Absorbances were measured in a plate spectrophotometer at 492 nm. The titers were defined as the highest dilution with an A_492_ ≥ 0.1 [34].

### 2.7. Hemagglutination Inhibition (HI) Assay

HI assay was performed with the sera collected at the end of the immunization schedule. Sera were treated with the receptor-destroying enzyme (RDE, Denka Seiken Co., Gosen-shi, Niigata-ken, Japan) to eliminate non-specific HI inhibitors at 37 °C for 18 h. Inactivation of the enzyme was performed for 30 min at 56 °C. HI-neutralizing antibodies in serum were assayed individually or in the pool for HI titers using a 1% guinea pig red blood cell (RBC) suspension, following a described protocol [35]. Titers were recorded as the reciprocal of the highest serum dilution that completely inhibited hemagglutination. The arithmetic mean of replicate results was used for analysis. The initial dilution was defined as 1:10; serum samples without detectable HI were assigned a titer of 5.

### 2.8. Microneutralization (MN) Assay

Virus MN assay was performed using a virulent wild type H5N1 clade 2.3.4.4b virus isolated from the carcass of an infected bird (Royal tern, *Thalasseus maximus*) collected on the coast of Espirito Santo State (Brazil) in June 2023, A/Thalasseus_maximus/Brazil-ES/23ES1A0008/2023 (H5N1), GISAID: EPI_ISL_18130597 [36]. The virus was replicated in chick embryo-related (CER) cells [37,38] kindly provided by Prof. Dr. H. M. Hafez (Free University of Berlin, Berlin, Germany). Briefly, CER cells were seeded on day 0 at a concentration of 3 × 10^3^ cells/well in a 96-well plate and maintained at 37 °C in a 5% CO_2_ incubator. On day 1, samples from the groups described in Table 1 were serial diluted using MEM culture medium, incubated with 100 µL of 100 TCID50 virulent H5N1 under agitation at 37 °C for 1 h. Subsequently, the supernatants were added to the CER cells on the 96-well plates and kept at 37 °C in a 5% CO_2_ incubator for 72 h. At the end of the incubation period, samples were observed under an inverted microscope and evaluated for the presence or absence of cytopathic effect (CPE). The last dilution at which no CPE was observed was considered the MN titer [39]. This assay was performed in a BSL-3 laboratory.

### 2.9. Statistical Analysis

H5Nx-specific hemagglutination inhibition titers and the microneutralization data were analyzed by one-way ANOVA followed by Tukey’s multiple comparison test using GraphPad Prism Software, version 5.03 for Windows. Significant differences were indicated (* *p* ≤ 0.05, ** *p* ≤ 0.01, *** *p* ≤ 0.001, **** *p* ≤ 0.0001).

## 3. Results

### 3.1. Selection of the Best CVVs Matching the Circulating H5N1 Viruses

**Figure 2:**
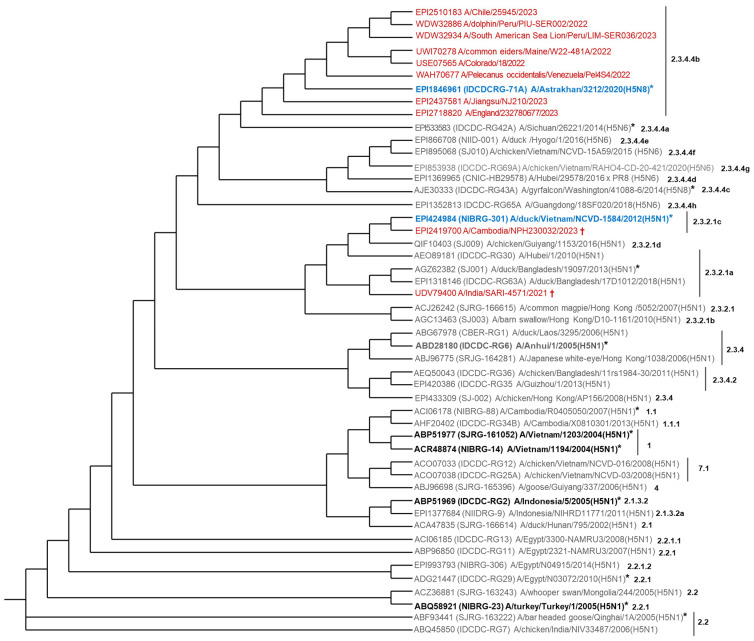
Phylogenetic relationship of some representative H5N1 circulating isolates combined with antigenic prototype H5Nx CVVs. The analysis was based on the H5 amino acid sequence. It was performed with 49 sequences (39 from the antigenic prototype of H5Nx CVVs and 10 from H5N1 isolates). In red, H5 sequences from six human isolates (GISAID: EPI2437581, EPI2510183, EPI2718820, EPI2419700; GenBank: USE07565, UDV79400); two non-human mammal isolates (GenBank: WDW32886, WDW32934); and two bird isolates (GenBank: UWI70278, WAH70677) (N = 10). In blue, the best CVVs for H5N1 clades 2.3.4.4b or 2.3.2.1c. In black and bold, FDA or EMA-licensed H5N1 vaccines (N = 4). In box, the three CVVs selected for vaccine developments at Butantan Institute. * CVVs with the potency testing reagents available. ^†^ fatal human case. The phylogenetic tree was created using the Interactive Tree of Life (iTOL) tool (https://itol.embl.de/, accessed on 28 May 2025).

Considering the lessons learned from past and recent pandemics, we must be prepared to respond to an H5N1 pandemic, especially in the case that this threat becomes real. We selected some representative H5N1 isolates of epidemiological and clinical relevance (two birds, two non-human mammals, and six humans) (see Supplementary Table S1). We compared their H5 amino acid sequence to the antigenic prototype of 39 H5Nx CVVs (31H5N1, 6H5N6 and 2H5N8) by phylogenetic analysis (Figure 2). Indeed, the H5 of the CVV IDCDC-RG71A A/Astrakhan/3212/2020 (H5N8) clustered with H5N1 isolates belonging to clade 2.3.4.4b circulating in North and South America (for simplicity, we will refer to this CVV as Astrakhan). The NIBRG-301 A/duck/Vietnam/NCVD-1584/2012 (H5N1) clustered with a clade 2.3.2.1c isolate (for simplicity, we will refer to this CVV as Duck/Vietnam). We also compared the H5 amino acid sequences of the globular domain between some isolates and the antigenic prototypes of CVVs with the potency testing reagents available (Supplementary Table S2), confirming that Astrakhan was the best CVV matching the predominant clade 2.3.4.4b of almost global distribution. Duck/Vietnam was also selected for clade 2.3.2.1c, responsible for the highest number of human fatal cases. Therefore, we decided to produce these two CVVs and a third one, IBCDC-RG6 A/Anhui/1/2005 (H5N1); for simplicity, we will refer to this CVV as Anhui. This CVV belongs to a more basal branch linking non-circulating as well as the currently circulating H5Nx (clades 2.3.2.1, 2.3.2.1a, 2.3.2.1b, 2.3.2.1c, 2.3.2.1d, 2.3.4.4a, 2.3.4.4b, 2.3.4.4c, 2.3.4.4d, 2.3.4.4e, 2.3.4.4f, 2.3.4.4g, 2.3.4.4h, and 2.3.4.2) (Figure 2). However, it has 86–89% amino acid sequence identity in the H5 globular domain with those of clades 2.3.4.4b, 2.3.2.1a, and 2.3.2.1c. The Anhui strain is antigenically closest to H5N1 clade 2.1.3.2 of a human-licensed H5N1 already approved for emergency use (CVV IDCDC-RG2 A/Indonesia/5/2005 (H5N1)); for simplicity, we will refer to this CVV as Indonesia (Figure 2, Supplementary Tables S2–S4, and Supplementary Figure S1). The decision to produce these three monovalent CVVs (Astrakhan, Duck/Vietnam, and Anhui) was also based on the existence of reference reagents used to calibrate the HA content [29,30]. Sequence alignments and amino acid differences were also analyzed and displayed in Supplementary Figure S1 and Tables S3 and S4.

### 3.2. Production of CVV Monovalent Bulks

We have produced the three monovalent CVV bulk concentrates (Astrakhan, Duck/Vietnam, and Anhui) selected as discussed above through current Good Manufacturing Practices (cGMP) at industrial scale, using the egg-based technology and the same infrastructure being used to produce the seasonal trivalent influenza vaccine (TIV) provided to the National Immunization Program to immunize the elderly and other population targets in Brazil [32]. Specific pathogen-free chicken embryonated eggs (SPF-CEE) have been used for working influenza virus bank, and controlled chicken embryonated eggs (controlled-CEE) have been used for the production of the monovalent antigens.

To obtain the working virus bank, we inoculated each CVV strain in 9-day-old SPF-CEE, and the allantoic liquid (AL) was collected after 24–72 h. Interestingly, the recovered embryos displayed hemorrhagic lesions when SPF-CEE were used (for producing the working virus bank), as observed in Supplementary Figure S2 for the Astrakhan, Duck/Vietnam, and Anhui viruses. These features were not observed with the controlled-CEE used for the production of the monovalent antigens (Astrakhan, Duck/Vietnam, and Anhui), nor with seasonal influenza CVV strains, in this last case, using SPF- or controlled-CEE (Supplementary Figure S2). The reasons for these differences are unknown.

Three lots of each monovalent concentrated bulk were produced, using 346,752 eggs/lot. The HA content was determined by the Single Radial Immunodiffusion (SRID) technique and the average concentration, doses/lot, and dose/egg (considering 15 µg HA/egg) were estimated. The estimated yield from the three CVVs produced is shown in Supplementary Table S5. The yields were distinct among the produced H5Nx concentrated monovalent antigens, depending on the CVVs, with the IDCDC-RG71A (A/Astrakhan/3212/2020 (H5N8)) CVV being of the lowest yield, and the NIBRG-301 (A/duck/Vietnam/NCVD-1584/2012 (H5N1)) CVV of the highest yield. All the produced monovalent CVVs were within the expected specifications (Supplementary Table S6).

### 3.3. Immunogenicity of Influenza CVV Formulations

**Figure 3:**
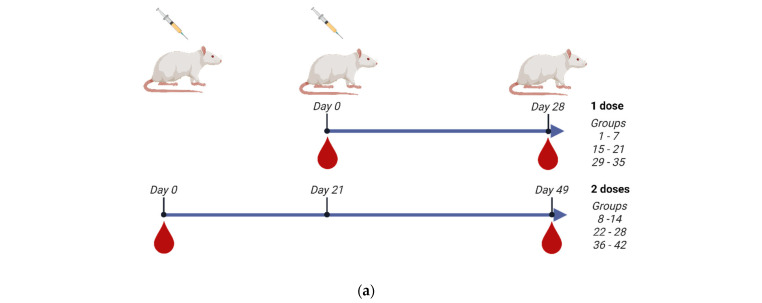

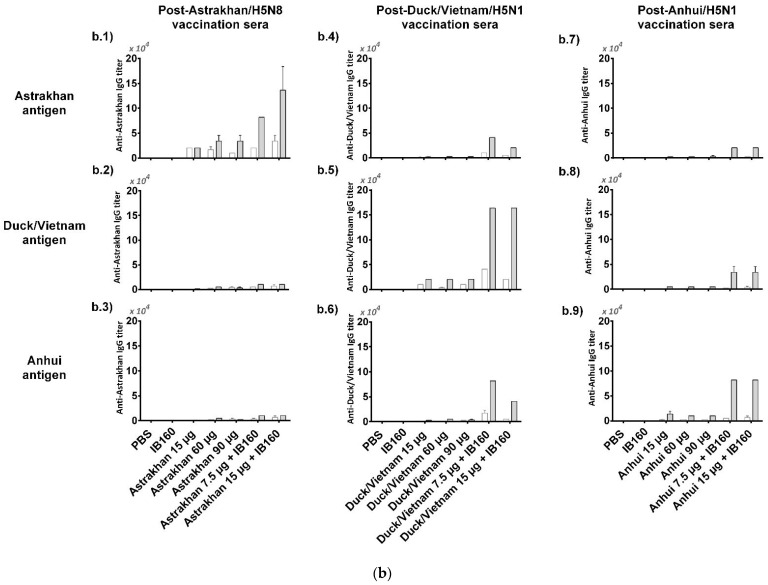

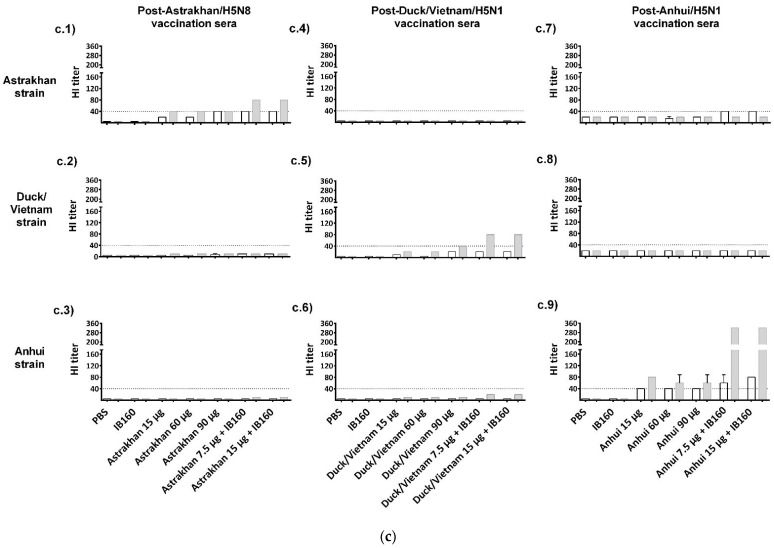
Immunogenicity of the three produced monovalent CVVs. The figure shows (**a**) the immunization schedule created with Biorender.com, (**b**) the specific anti-CVV IgG titer measured by ELISA, and (**c**) the titers of HI antibodies measured by HI assay using pools of animal sera. Groups of rats were immunized with one (white bars, N = 5) or two doses (gray bars, N = 6) of the vaccine formulations intramuscularly according to Table 1. (**b**) The antibodies elicited by different formulations against each CVV were assayed for the homotypic immune response (using the same strain immunized as coating) and for the heterotypic immune response (using one of the two remaining strains as coating). The bars represent the arithmetic mean of IgG antibody titer in pooled sera and the standard deviation among three replicates. (**c**) The antibodies elicited by different formulations against each CVV were assayed for the homotypic immune response (using the same strain immunized as hemagglutinating antigen) and for the heterotypic immune response (using one of the two remaining strains as hemagglutinating antigen). The bars represent the arithmetic mean of HI titer in pooled sera and the standard deviation between two replicates. The initial dilution was defined as 1:10; serum samples without detectable HI were assigned a titer of five. The horizontal dashed line represents the seroconversion threshold (when HI titers are equal to or greater than 1:40, these sera are considered seroconverted).

In order to evaluate the immunogenicity of the three monovalent CVV antigens, we have formulated vaccines with high content of HA as well as adjuvanted with IB160, a squalene oil-in-water emulsion adjuvant [26,40]. Supplementary Figure S3 shows the SDS-PAGE characterization of the monovalent antigens used in the vaccine formulations, showing a similar profile among the produced CVVs. The vaccine formulations were well tolerated and the vaccinated animals did not show signs of vaccine toxicity (Figure S4), as evaluated by animal weight gains. For the immunogenicity studies, 60 or 90 µg of HA/dose were tested and compared with the usual 15 µg of HA as formulated in the seasonal TIV vaccines. The 15 µg of HA was also compared to 7.5 or 15 µg of HA adjuvanted with IB160. We have also analyzed one- (groups 1–7, 15–21, and 29–35 for Astrakhan, Duck/Vietnam, and Anhui, respectively) or two-dose (groups 8–14, 22–28, and 36–42 for Astrakhan, Duck/Vietnam, and Anhui, respectively) immunization protocol (Table 1, Figure 3a). In the case of the two-dose immunization protocol, the animals were immunized with a 21-day interval between each immunization. The animal bloods were collected 28 days after the last dose for total IgG titer in Enzyme-Linked Immunosorbent Assay, hemagglutination inhibition assay, or microneutralization assay in H5N1 permissive cells.

We assessed the homotypic and heterotypic immune response against the three monovalent antigens produced (Astrakhan, Duck/Vietnam, and Anhui). The serum of each animal from each group was combined to constitute a pool of sera for a general view of the immune response generated by the vaccine formulations. We can observe the titers of total IgGs obtained after one or two doses (Figure 3b). Compared to negative control animal groups immunized with PBS or adjuvant IB160, all the groups of animals that received the different formulations with the antigens derived from each virus CVV produced detectable anti-CVV IgG antibodies. This response was specific against the homologous strain and detectable in the lowest dose (15 µg HA alone). For instance, all the groups of animals that received Astrakhan antigens produced detectable specific anti-Astrakhan antibodies (Figure 3(b.1)), even after the first immunization and in the lowest dose (15 µg HA alone). Increasing the dose, the titers in one-dose regimen immunization (60 and 90 µg of HA) were not increased. After two doses, the antibody titers increased for every animal group immunized with Astrakhan antigens except the animal group immunized with 15 µg of HA. Interestingly, the titers of specific antibodies against Astrakhan antigens in animal groups immunized with 15 µg of Astrakhan antigens adjuvanted with IB160 after one dose were similar to those animal groups immunized with 60 or 90 µg HA after two doses. However, after the second immunization, the superiority of the group of animals immunized with Astrakhan antigens adjuvanted with IB160 becomes evident. The highest titer was obtained in the animals immunized with two doses of 15 µg of HA with IB160. Based on these results, adjuvanted Astrakhan antigens seemed to provide a better immune response measured by specific antibodies against the homologous antigen. However, these antibodies do not react well with heterologous antigens like Duck/Vietnam or Anhui (Figure 3(b.2,b.3)). Similar responses were observed when sera from animals immunized with Duck/Vietnam (Figure 3(b.4–b.6)) or Anhui (Figure 3(b.7–b.9)) vaccine formulations were tested, showing a clade-specific humoral immune response, though some cross-reactivity against heterologous antigens were observed in sera of animals immunized for Duck/Vietnam and Anhui vaccine formulations in the two-dose protocol, particularly when the monovalent antigens were formulated with IB160 adjuvant. These results show that IB160 is an important adjuvant to be used in combination with these H5Nx antigens and in two immunization protocols, which result in a more robust production of specific antibodies against H5Nx antigens.

We also examined the ability of these antibodies in the pooled sera to inhibit hemagglutination induced by each CVV (Figure 3c). HI titers equal or higher than 1:40 are generally recognized as corresponding to a 50% reduction in the risk of influenza [41]. We can observe that the animal groups immunized with high-dose and adjuvanted Astrakhan antigens (90 µg, 7.5 µg + IB160, and 15 µg + IB160) achieved 1:40 titers after one immunization, unlike other immunization groups. However, the HI titers doubled in the animal groups immunized with IB160 adjuvanted Astrakhan antigens after the second dose (Figure 3(c.1)). In general, the same trend was observed for the animals immunized with the other H5Nx, making the two-dose regimen using the antigens combined with adjuvant IB160 the best condition to achieve higher HI titers. Cross-HI inhibition with heterologous viruses was not observed. The highest HI activity was observed in the Anhui vaccine formulations when compared with Astrakhan and Duck/Vietnam.

**Figure 4:**
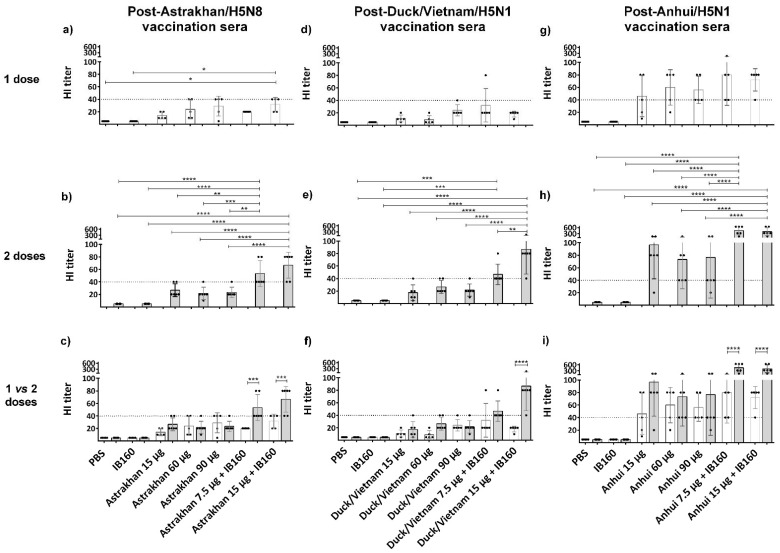
H5Nx-specific hemagglutination inhibition titer of individual serum of animals immunized with one or two doses of vaccine formulations. The presence of specific antibodies in sera of individual animals immunized by vaccine formulations used to evaluate the immunogenicity of each CVV (Astrakhan, Duck/Vietnam, or Anhui)-derived formulation, capable of inhibiting red blood cell hemagglutinations by the corresponding homologous virus strain, was titrated. The sera from animals immunized with formulations of the three monovalent bulks comprising split and inactivated H5Nx antigens were collected 28 days after the last dose of the immunization schedules: one-dose (white bars, N = 5) and two-dose (gray bars, N = 6). The individual serum was from the animals of the same assay as described in Figure 3. The bars represent the arithmetic mean among the animals and the standard deviation. Each black circle symbol represents one animal per group. The horizontal dashed line represents the HI titer threshold; equal or higher than 1:40 is considered seroconverted. Panels (**a**–**c**) show hemagglutination induced by Astrakhan CVV and sera evaluated from individual animals immunized with Astrakhan antigen formulations; panels (**d**–**f**) show hemagglutination induced by Duck/Vietnam CVV and sera evaluated from individual animals immunized with Duck/Vietnam antigen formulations; and panels (**g**–**i**) show hemagglutination induced by Anhui CVV and sera evaluated from individual animals immunized with Anhui antigen formulations (see Table 1). Comparing the vaccination effect between various formulations, an asterisk * indicates a significant difference by one-way ANOVA (* *p* ≤ 0.05, ** *p* ≤ 0.01, *** *p* ≤ 0.001, **** *p* ≤ 0.0001).

Since the HI was also clade-specific, we next analyzed in more detail the HI titers of the individual animals against hemagglutination induced by its homologous virus, as shown in Figure 4. To facilitate the statistical analysis, we compared the immune response among the vaccine formulations of animals immunized with only one dose (Figure 4a,d,g) or two doses (Figure 4b,e,h) or between one versus two doses (Figure 4c,f,i). It was observed that in the one-dose regimen, the immune responses were very poor and not statistically significant, except for Astrakhan in animals immunized with 15 µg of Astrakhan monovalent antigen combined with IB160 when compared to negative control animals immunized with PBS or IB160 (Figure 4a,d,g). However, with two doses, all the animals immunized with 7.5 or 15 µg combined with IB160 were similar among each other and significantly different from all the other immunized groups in the case of vaccine formulations with Astrakhan and Anhui antigens (Figure 4b,h). In the case of Duck/Vietnam, HI titers from animals immunized against 15 µg combined with IB160 were different from all the other animal groups. The HI titers from animals immunized with 7.5 µg combined with IB160 were significantly different only from the negative control animals immunized with PBS or IB160 (Figure 4e) in the two-dose protocol. When we compare the one-dose versus two-dose regimen within each animal group, we can observe that in all animal groups immunized with 15 µg of H5Nx (Astrakhan, Duck/Vietnam or Anhui) monovalent antigens with IB160, two doses elicited a significantly higher antibody response capable of HI when compared with one immunization; the same was true for Astrakhan and Anhui in the case of animals from the group vaccinated with 7.5 µg of antigen combined with IB160. The same group in the case of Duck/Vietnam did not present differences in HI titers between the first and second doses (Figure 4c,f,i).

### 3.4. Seroneutralization of Brazilian Wild-Type H5N1 Virus Clade 2.3.4.4b

H5N1 viruses from clade 2.3.4.4b are spreading all over the world with the exception of Australia and New Zealand. The capacity of the sera of immunized animals with formulated vaccines with Astrakhan antigens to inhibit infection by wild-type H5N1 virus was analyzed. This virus was isolated in infected birds in Brazil and belongs to clade 2.3.4.4b [36], the same as Astrakhan CVV.

**Figure 5:**
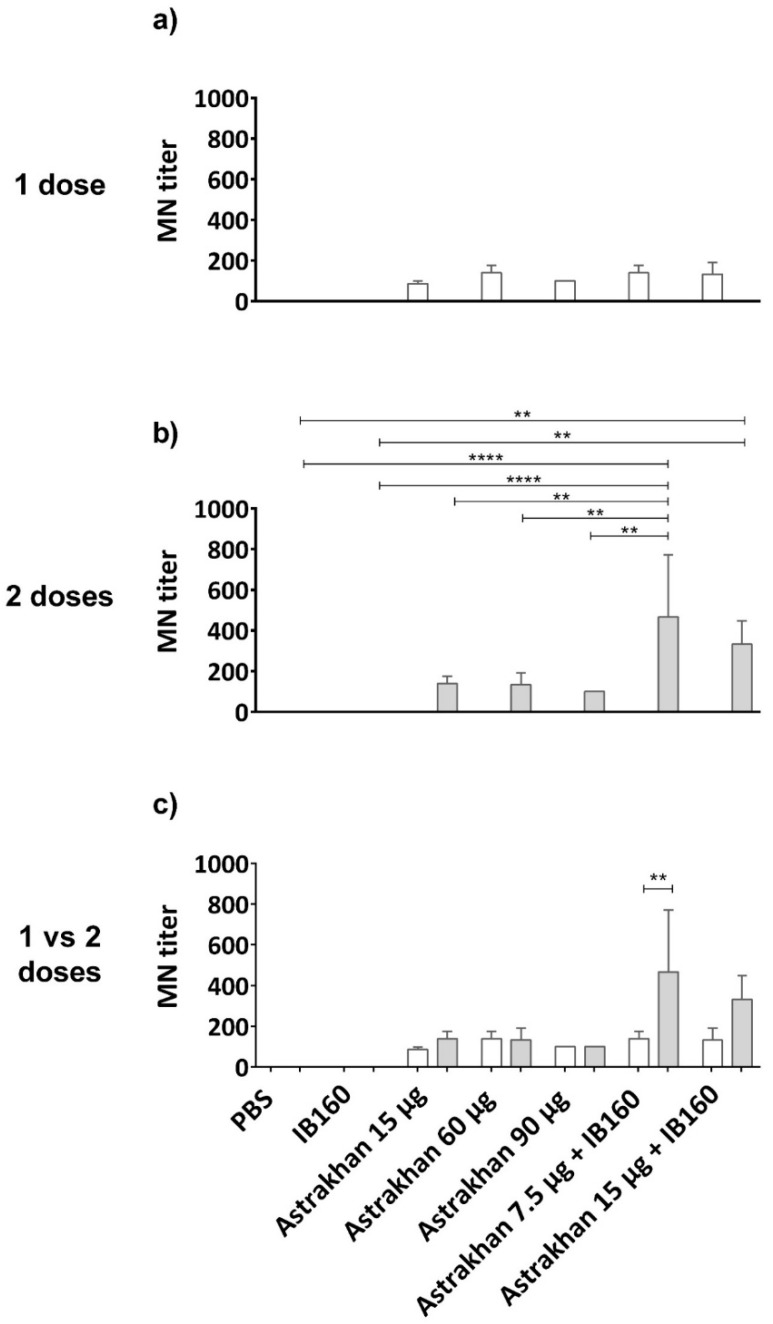
Microneutralization (MN) titers elicited by the pool of serum from immunized animals with Astrakhan (H5N8) CVV formulations. Rats were immunized (**a**,**c**) once (white bars, N = 5) or (**b**,**c**) twice (gray bars, N = 6) with different formulations of Astrakhan inactivated vaccines with or without IB160 adjuvant (Figure 3a). The MN assay was performed against a wild-type HPAI H5N1 virus using samples of pooled sera collected at day 28 post the last immunization of each group (see Table 1). The pooled sera used were from the same assay as described in Figure 3. CER cells were infected with 100 TCID50 of the wild-type H5N1 virus pre-incubated with serial dilutions of pooled sera for 1 h at 37 °C. The MN titer was considered the highest serum dilution at which no cytopathic effect (CEP) was observed, indicating no infection. The bars represent the arithmetic mean of the titer of three replicates of the MN assay. ** or **** indicates a significant difference by one-way ANOVA (*p* ≤ 0.01 and *p* ≤ 0.0001, respectively). The figure shows the comparison of the pooled sera titer (**a**) among experimental groups on a one-dose immunization schedule, (**b**) among experimental groups on a two-dose immunization schedule, and (**c**) between one and two doses for each experimental group. In panel (**c**), the MN titer of pooled sera from animals immunized with two doses of 7.5 µg of HA + IB160 is also significantly different from the titers of pooled sera from the remaining animal groups, except for two doses of 15 µg HA + IB160.

A microneutralization (MN) assay was performed on permissive CER cells (Figure 5). The results showed that Astrakhan (H5N8) vaccines could induce neutralizing antibodies against this wild-type HPAI H5N1 bird isolate related to clade 2.3.4.4b [36]. The levels of neutralizing antibodies were not antigen dose-dependent in non-adjuvanted vaccine formulations. Moreover, the titers were similar after immunization with one or two doses of non-adjuvanted vaccines (Figure 5c). On the other hand, formulation of Astrakhan antigens with IB160 adjuvant and in two immunizations more than doubled MN titers at dose 7.5 µg HA + IB160, when comparing one- and two-dose regimens as well as the two doses with all the other vaccine formulations, with the exception of two doses of 15 µg HA + IB160 (Figure 5b,c). In two dose immunization, 15 µg HA + IB160 elicited higher HI titers than negative controls (PBS and IB160), though they were not significantly different from non-adjuvanted antigens. One dose of 7.5 µg HA + IB160 had titers similar to non-adjuvanted (either in one or two administrations) (Figure 5a,b). Altogether, these results indicate the importance of a two-dose regimen and the use of IB160 adjuvant in vaccine formulations using Astrakhan against H5 clade 2.3.4.4b.

## 4. Discussion

Since the last pandemic, the scientific community and government agencies have learned that vaccines are the first and most important step in protecting against influenza viruses [42]. As part of countermeasures for pandemic preparedness, it is necessary to maintain stockpiles of efficient candidate vaccines to be delivered in the shortest time for use by the population. The CVVs available for the predominant H5 2.3.4.4b clade are IDCDC-RG71A and CBER-RG8A, both derived from the antigenic prototype A/Astrakhan/3212/2020 (H5N8) and IDCDC-RG78A, derived from A/American wigeon/South Carolina/22-000345-001/2021-like (H5N1); the CVV for the 2.3.2.1c clade is NIBRG-301 (A/duck/Vietnam/ (H5N1)) [29,30]. Our strategy was to select the best CVV matching the predominant and overspread H5 2.3.4.4b clade (Astrakhan) and also a more restricted but lethal clade, H5 2.3.2.1c (Duck/Vietnam). We have also included the IBCDC-RG6 (A/Anhui/1/2005 (H5N1)) CVV, which is in a more basal position than Astrakhan and Duck/Vietnam and also closest to the H5N1 registered vaccines for emergency use that could also be considered as vaccine candidates (Figure 2). These CVVs, as well as the reference reagents, are available for the production and calibration of these three monovalent CVVs (Astrakhan, Duck/Vietnam, and Anhui) through the WHO Collaborating Centers [29,30].

Three lots of each monovalent antigen were produced in an egg-based industrial seasonal TIV plant to evaluate their yields. As expected, they vary according to the CVV used for production. This information can be used for production planning in case of necessity (Table S5).

In the case of H5N1 clade 2.3.4.4b, the virus is evolving rapidly and is now of almost global distribution except in Australia and New Zealand [1]. It is easily spread by birds throughout the continents and regions, affecting not only wild but also urban pet animals, like cats, and animal livestock for human consumption, like dairy cattle, alpacas, goats, pigs, poultry, and minks, with some occasional human infections through contact with infected animals [11,43,44]. This indicates the ability of bird-to-bird, bird-to-mammal, bird-to-human, mammal-to-mammal, and mammal-to-human transmissions. Fortunately, no human-to-human transmission has been observed [44]. Many changes have been observed in the genomes of some related virus isolates that may facilitate mammal transmission, replication, and virulence, like the PB2-E627K, PB2-D701N, and PB2-T271A mutations [45]. In addition, the events observed in gain-of-function experiments—described in 2012 for the H5N1 virus [46,47]—may occur through natural evolution processes, resulting in mutations and amino acid changes that resemble those intentionally performed in the gain-of-function studies. The viral HA mediates virus binding to cellular receptors and is the first virus protein that helps to define host range restriction and susceptibility. Only four mutations in this protein were enough to increase respiratory droplets and air transmissibility to ferrets in a reassorted H5 with the 2009 pandemic H1N1 or in a HPAI H5N1 A/Indonesia/5/2005, after a few animal passages. In the last case, an additional amino acid change in the PB2 protein was included to result in a virus with high transmissibility. Currently, it was demonstrated using the ferret model that a mink-derived H5N1 isolate transmitted to 75% of ferrets exposed by direct contact. In airborne transmission studies, the virus transmitted to 37.5% of respiratory contact animals developing an infection [48]. Ocular transmission was also observed in this animal model, causing severe and fatal infections in ferrets [49]. Although the pandemic risk continues to be evaluated as low, the current circulation of H5N1 in dairy cows has been causing sporadic infections in farm workers, raising public health concerns [44]. The virus isolated from a Texas worker (huTX37-H5N1), who experienced only mild symptoms, caused fatal infections in mice and ferrets, spreading systemically and reaching high virus titers in respiratory and non-respiratory organs. Additionally, in four independent experimental studies in ferrets, huTX37-H5N1 could be transmitted through respiratory droplets and cause fatal infections [50]. The recent increase in human cases in the USA and Canada is of concern (Figure 1) and suggests the presence of two genotypes, one associated with mild cases and derived mostly from dairy cattle (genotype B3.13) and the other one associated with severe cases and derived from birds and poultry (genotype D1.1) [19,51,52]. Indeed, one fatal human case associated with genotype D1.1 was just reported in Louisiana (USA) [53]. However, no direct correlation between pathogenicity and viral genotype has been proven. Altogether, these data indicate that the circulating H5N1 clade 2.3.4.4b virus is evolving and can reach a high transmissibility and adaptability of mammal-to-mammal, mammal-to-human, and human-to-human infections due to few mutations in the genome [50,54]. In addition, considering the CVVs and the potency testing reagents available [25,29], Astrakhan seems to be very suitable for vaccine formulations against clade 2.3.4.4b based on HA sequence. Though IDCDC-RG78A (A/American wigeon/South Carolina/22- 000345-001/2021-like (H5N1)) CVV is derived from an A/H5N1 isolate, in contrast to Astrakhan, which originates from an A/H5N8 isolate, the sequence identity of the HA globular domain—which interacts with the cellular receptor—from Astrakhan is 100% identical to that of the H5 clade 2.3.4.4b genotype D1.1, whereas IDCDC-RG78A shows 99.14% of identity (Tables S7 and S8, Figure S5). As mentioned above, genotype D1.1 is associated with mild to severe infections and is also linked to a fatal case [19,51,52].

Our data showed that the immune responses with vaccines formulated with Astrakhan, Duck/Vietnam, or Anhui are very specific to their homologous viruses and antigens. Therefore, the already registered H5N1 vaccines for emergency use against those initial strains are not expected to protect against the current circulating viruses. These vaccines have to be updated with the new circulating strains. Other reports reached the same conclusion as discussed above concerning the specificity of the vaccines and the lack of cross-reactivity and protection [17,55]. However, recent results reported that the stockpiled vaccines that belong to distinct clades can elicit neutralizing antibodies against H5N1 clade 2.3.4.4b [56]. More data will be needed to clarify these discrepancies. For the circulating clades 2.3.4.4b and 2.3.2.1c, it seems that it is necessary to have two immunizations and that the best formulations were those comprising the antigens combined with adjuvant IB160. Neither 90 nor 60 µg used as a high dose showed the same performance compared to the adjuvanted formulations, which also have the advantage of sparing the doses, increasing the productivity by six to twelve times when compared to the high dose formulations. This can be an important issue in case a pandemic occurs. IB160 belongs to a class of adjuvants that contains squalene in an oil-in-water emulsion [57,58]. Interestingly, 90, 60, or 15 µg of the antigens alone elicited similar low HI titers in one- or two-immunization protocols, in contrast to adjuvanted 7.5 or 15 µg with IB160 in the two-dose protocol. All the adjuvanted formulations of each produced monovalent antigen induced HI antibodies with titers equal to or greater than 1:40. Serum hemagglutinin inhibition antibody titers of 40 are correlated with a 50% protection from clinical disease caused by seasonal influenza viruses in humans [41]. Because the correlates of protection against H5N1 HPAI have not been established to date, the implications of our findings will require further investigation. In summary, similar to vaccine formulations against H7N9, the H5N1 vaccine antigen is a poor immunogen. It requires two doses and the adjuvant IB160, which can be replaced by similar squalene-based adjuvants [26].

Preparedness for a potential pandemic is a lesson learned from recent times. The lack of sufficient doses of vaccines resulted in higher mortality, especially in those countries that lack enough resources to purchase the vaccines, like in the case of COVID-19. Even when the countries have these resources, they may wait in line until the production is sufficient to deliver the vaccines. In the meantime, the population will be at risk, and preventable deaths through vaccination will be feasible. mRNA vaccines are in development [59] and may be an important tool in case of an influenza H5N1 pandemic. However, the current egg-based seasonal influenza vaccine manufacturers may also have an important contribution if an H5N1 pandemic occurs.

## 5. Conclusions

The results shown here are a step forward to provide a rapid local response in case of the occurrence of a H5N1 pandemic influenza in Brazil, which will also affect the neighboring countries of South America. Moreover, it also shows the increased capability of a global response against a pandemic infectious disease, complementing egg-based vaccines as well as vaccines produced by other platforms, such as RNA vaccines and cell-based vaccines. Considering the CVVs with the testing potency reagents available and that the antibody response obtained against the CVVs produced was clade-specific, IDCDC RG-71A is the indicated CVV for the predominant currently circulating H5N1 influenza virus of 2.3.4.4b clade. This CVV must be combined with adjuvant to induce a higher and efficacious immune response in a two-dose immunization protocol. For the H5N1 clade 2.3.4.4b, which is well adapted and highly spread with the potential to evolve into a more infectious virus, our results are promising enough to proceed with the next steps of vaccine development to evaluate safety and immunogenicity in clinical studies.

## Figures and Tables

**Table 1:** Experimental groups.

Formulations’ Abbreviated Codes							
A/Astrakhan/3212/2020 (H5N8) [IDCDC-RG71A]		A/duck/Vietnam/NCVD-1584/2012 (H5N1) [NIBRG-301]		A/Anhui/1/2005 (H5N1) [IBCDC-RG6]		Number of Doses	Number of Animals/ Group
Groups	Astrakhan	Groups	Duck/Vietnam	Groups	Anhui		
1	PBS	15	PBS	29	PBS	1	5
2	IB160	16	IB160	30	IB160	1	5
3	Astrakhan 15 µg	17	Duck/Vietnam 15 µg	31	Anhui 15 µg	1	5
4	Astrakhan 60 µg	18	Duck/Vietnam 60 µg	32	Anhui 60 µg	1	5
5	Astrakhan 90 µg	19	Duck/Vietnam 90 µg	33	Anhui 90 µg	1	5
6	Astrakhan 7.5 µg + IB160	20	Duck/Vietnam 7.5 µg + IB160	34	Anhui 7.5 µg + IB160	1	5
7	Astrakhan 15 µg + IB160	21	Duck/Vietnam 15 µg + IB160	35	Anhui 15 µg + IB160	1	5
8	PBS	22	PBS	36	PBS	2	6
9	IB160	23	IB160	37	IB160	2	6
10	Astrakhan 15 µg	24	Duck/Vietnam 15 µg	38	Anhui 15 µg	2	6
11	Astrakhan 60 µg	25	Duck/Vietnam 60 µg	39	Anhui 60 µg	2	6
12	Astrakhan 90 µg	26	Duck/Vietnam 90 µg	40	Anhui 90 µg	2	6
13	Astrakhan 7.5 µg + IB160	27	Duck/Vietnam 7.5 µg + IB160	41	Anhui 7.5 µg + IB160	2	6
14	Astrakhan 15 µg + IB160	28	Duck/Vietnam 15 µg + IB160	42	Anhui 15 µg + IB160	2	6

## Data Availability

The data that support the findings of this study are openly available in the repository “Characterization of influenza H5Nx Candidate Vaccine Virus formulations” at https://doi.org/10.6084/m9.figshare.27829143. Data will be made available upon request through contact with the corresponding author, with an appropriate data-sharing agreement.

## Supplementary Materials

The following supporting information can be downloaded at: https://www.mdpi.com/article/10.3390/vaccines13060620/s1. The supplementary information file contains Table S1: H5N1 influenza virus isolates selected for BLAST and phylogenetic analysis; Table S2: Identity of the H5 hemagglutinin globular domain of influenza H5N1 virus circulating in the Americas and Asia to antigenic prototypes of CVVs with available potency testing reagents and H5N1 licensed vaccines; Table S3: Identity of H5 hemagglutinin among CVVs produced at Butantan Institute (Astrakhan, Duck/Vietnam, and Anhui) and CVV Indonesia; Figure S1: Multiple Sequence Alignment of the H5 amino acid sequences of the H5Nx CVVs produced at Butantan Institute (Astrakhan, Duck/Vietnam, and Anhui) and CVV Indonesia; Table S4: Different amino acids among produced CVVs (Astrakhan, Duck/Vietnam, and Anhui) and Indonesia CVV; Figure S2: Embryos recovery after infection with H5Nx CVVs; Table S5: Estimated yields for H5Nx vaccines; Table S6: Monovalent bulks produced for each CVV were within defined specifications; Figure S3: SDS-PAGE of allantoic liquid (AL) and final split H5Nx vaccine antigens (Mono) from A/Astrakhan/3212/2020 (H5N8), A/Anhui/1/2005 (H5N1), and A/duck/Vietnam/NCVD-1584/2012 (H5N1) CVVs; Figure S4: Weight loss/gain curves of rats immunized with the H5Nx vaccine formulations; Table S7: Identity of H5 hemagglutinin among CVVs belonging to clade 2.3.4.4b and American H5N1 isolates; Figure S5: Multiple Sequence Alignment of the H5 amino acid sequences among 2.3.4.4b H5Nx CVVs and H5N1 isolates; and Table S8: Different amino acids among H5 sequences of 2.3.4.4b CVVs and H5N1 isolates. Separate graphics and images of the combined supplementary figures can also be found in this repository.
