# Supplementary material for: Production and Immune Response Against Pandemic Influenza Candidate Vaccines as Preparedness Against the Circulating H5N1 Influenza Viruses
Source: Vaccines (Basel). 2025 Jun 8;13(6):620. doi: 10.3390/vaccines13060620 (PMC12197369; doi:10.3390/vaccines13060620)
Supplement: Supplementary file 1 [file vaccines-13-00620-s001.zip › vaccines-3645367-supplementary.pdf]

SUPPLEMENTARY INFORMATION

1. Selection of the best Candidate Vaccine Viruses (CVVs) matching isolates of clinical and epidemiological relevance

BLAST and phylogenetic analysis (see Figure 2, manuscript) of ten H5 sequences of circulating H5N1 isolates (Table S1) and H5Nx CVVs with potency testing reagents available (Table S2). The analysis allowed selecting the best CVV matching the HPAI H5N1 circulating virus from clades 2.3.2.1a, 2.3.2.1c, and 2.3.4.4b.

Table S1. **H5N1 influenza virus isolates selected for BLAST and phylogenetic analysis** (see Figure 2, manuscript).

Origin	Clade	Name	Isolate ID	H5 amino acid sequence ID	Clinical and epidemiological data
Human	2.3.2.1a	A/India/SARI-4571/2021(H5N1)	OL311387.1	UDV79400	Collected in July, 2021 in New Delhi, India, 11-year-old male, DIED.
	2.3.2.1c	A/Cambodia/NPH230032/2023(H5N1)	EPI_ISL_17024123	EPI2419700	Collected on February 21st, 2023 in Cambodia, 11-year-old female DIED.
	2.3.4.4b	A/Colorado/18/2022 (H5N1)	ON759331.1	USE07565	Collected on April 20th, 2022 in United States, survived.
		A/England/232780677/2023(H5N1)	EPI_ISL_18161874	EPI2718820	Collected on July 7th, 2023 in United Kingdom, survived.
		A/Jiangsu/NJ210/2023(H5N1)	EPI_ISL_17075747	EPI2437581	Collected on February 10th, 2023 in China, hospitalized, survived.
		A/Chile/25945/2023(H5N1)	EPI_ISL_17468386	EPI2510183	Collected on March 24th, 2023, 53 year-old-male, survived.
Non-human mammal	2.3.4.4b	A/dolphin/Peru/PIU-SER002/2022(H5N1)	OQ550442.1	WDW32886	Collected on November 22nd, 2022 in Peru.
		A/South American Sea Lion/Peru/LIM-SER036/2023(H5N1)	OQ550474.1	WDW32934	Collected on January 23rd, 2023 in Peru.
Bird	2.3.4.4b	A/common eiders/Maine/W22-481A/2022(H5N1)	OP377598.1	UWI70278	Collected on June 12nd, 2022 in United States.
		A/Pelecanus occidentalis/Venezuela/Pel4S4/2022(H5N1)	OP950305.1	WAH70677	Collected on November 25th, 2022, Venezuela.

Table S2. Identity of the H5 hemagglutinin globular domain of influenza H5N1 virus circulating in the Americas and Asia to antigenic

prototypes of CVVs with available potency testing reagents and H5N1 licensed vaccines.

Candidate Vaccine Virus	Antigenic prototype	Clade	H5 sequence ID[b]	Identity (%)[a]			
				UWI70278[c] (2.3.4.4b)	UDV79400[d] (2.3.2.1a)	EPI2419700[d] (2.3.2.1c)	EPI2510183[d] (2.3.4.4b)
NIBRG-14**[I]	A/Vietnam/1194/2004 (H5N1)	1	ACR48874.1	84.55	87.98	87.12	84.98
SJRG-161052**[II]	A/Vietnam/1203/2004 (H5N1)	1	ABP51977	84.98	88.41	87.55	85.41
NIBRG-88*	A/Cambodia/R0405050/2007 (H5N1)	1.1	ACI06178	85.84	88.41	87.12	86.27
IDCDC-RG2**[III]	A/Indonesia/5/2005 (H5N1)	2.1.3.2	ABP51969.1	84.55	89.27	87.12	84.98
SJRG-163222*	A/bar headed goose/Qinghai/1A/2005 (H5N1)	2.2	ABP93441	84.55	87.55	87.12	84.98
NIBRG-23**[IV]	A/turkey/Turkey/1/2005 (H5N1)	2.2.1	ABQ58921	84.12	87.12	86.70	84.55
IDCDC-RG29*	A/Egypt/N03072/2010 (H5N1)	2.2.1	ADG21447	82.40	85.41	85.41	82.84
SJ001*	A/duck/Bangladesh/19097/2013 (H5N1)	2.3.2.1a	AGZ62382	85.84	95.71	92.28	85.84
**NIBRG-301***	**A/duck/Vietnam/NCVD-1584/2012 (H5N1)**	2.3.2.1c	EPI424984	84.98	91.85	95.71	85.41
**IDCDC-RG6***	**A/Anhui/1/2005 (H5N1)**	2.3.4	ABD28180.1	86.70	89.70	86.67	87.12
IDCDC-RG42A*	A/Sichuan/26221/2014 (H5N6)	2.3.4.4a	EPI533583	95.28	88.41	83.69	94.85
**IDCDC-RG71A***	**A/Astrakhan/3212/2020 (H5N8)**	2.3.4.4b	EPI1846961	99.14	87.98	84.12	98.71
IDCDC-RG43A*	A/gyrfalcon/Washington/41088-6/2014 (H5N8)	2.3.4.4c	AJE30333.1	94.42	87.12	83.69	93.99

[a] Identity of the globular domain delimited by the conserved Cys 58 and 290 residues (H5 numbering); [b] H5 sequence access number of the antigenic prototype; [c] bird isolate; [d] human isolate; *potency testing reagents available; **FDA or EMA licensed vaccine: [I] GlaxoSmithKline Biologicals, [II] Sanofi Pasteur Inc, [III] ID Biomedical Corporation of Quebec, and [IV]AUDENZ-Seqirus. GenBank IDs: UWI70278 (A/common eiders/Maine/W22-481A/2022 (H5N1)) and UDV79400 (A/India/SARI-4571/2021 (H5N1)); GISAID EpiFlu IDs: EPI2419700 (A/Cambodia/NPH230032/2023 (H5N1)) and EPI2510183 (A/Chile/25945/2023 (H5N1)). The amino acid sequence of the three CVV antigens produced at Butantan Institute are in bold. The highest amino acid sequence identities of the circulating isolates to the corresponding CVVs are underlined. Observations: SJ001 CVV belongs to the same clade as UDV79400; NIBRG-301 (in bold) CVV belongs to the same clade as EPI2419700; IDCDC-RG71A (in bold) CVV belongs to the same clade as UWI70278 and EPI2437581.

The CVV IDCDC-RG71A (A/Astrakhan/3212/2020 (H5N8)) is referred as Astrakhan, the CVV NIBRG-301 (A/duck/Vietnam/NCVD-1584/2012 (H5N1)) as Duck/Vietnam, and the CVV IBCDC-RG6 (A/Anhui/1/2005(H5N1)) as Anhui for simplicity (Table S2).

BLAST analysis of the H5 amino acid sequences of the three monovalent antigens produced (Astrakhan, Duck/Vietnam, and Anhui) and the CVV IBCDC-RG2 (A/Indonesia/05/2005(H5N1)) was also performed (Table S3). The latter CVV belonging to clade 2.1.3.2 is the human licensed H5N1 vaccine antigenically closest to the predominant circulating H5 2.3.4.4b clade (see Figure 2, manuscript), and it was denominated as Indonesia in Table S3 and Figure S1 for simplicity.

Table S3. Identity of H5 hemagglutinin among CVVs produced at Butantan Institute (Astrakhan, Duck/Vietnam, and Anhui) and CVV Indonesia.

	Identity[a] (%)			
**CVV**	**Astrakhan**	**Duck/ Vietnam**	**Anhui**	**Indonesia**
**Astrakhan**	100	85.84	87.12	85.41
**Duck/Vietnam**	85.84	100	88.84	88.41
**Anhui**	87.12	88.84	100	93.99
**Indonesia**	85.41	88.41	93.99	100

A/Astrakhan/3212/2020(H5N8), GenBank: UJS29065; A/duck/Vietnam/NCVD-1584/2012(H5N1), GISAID EpiFlu: EPI1255841; A/Anhui/1/2005(H5N1), GenBank: AXN59219; A/Indonesia/5/2005(H5N1), GenBank: ABP51969. [a] identity of the globular domain delimited by the conserved Cys 58 and 290 residues (H5 numbering). Observation: H5 amino acid sequence access numbers of the CVV with the polybasic cleavage site modified.

Multiple sequence alignment among them is shown in Figure S1. Different amino acids among them are listed in Table S4, as well as the position and domain in the protein. Comparing the three CVVs produced it was found fifty-four positions with different amino acids: 11 in the stalk and 43 in the globular domain.

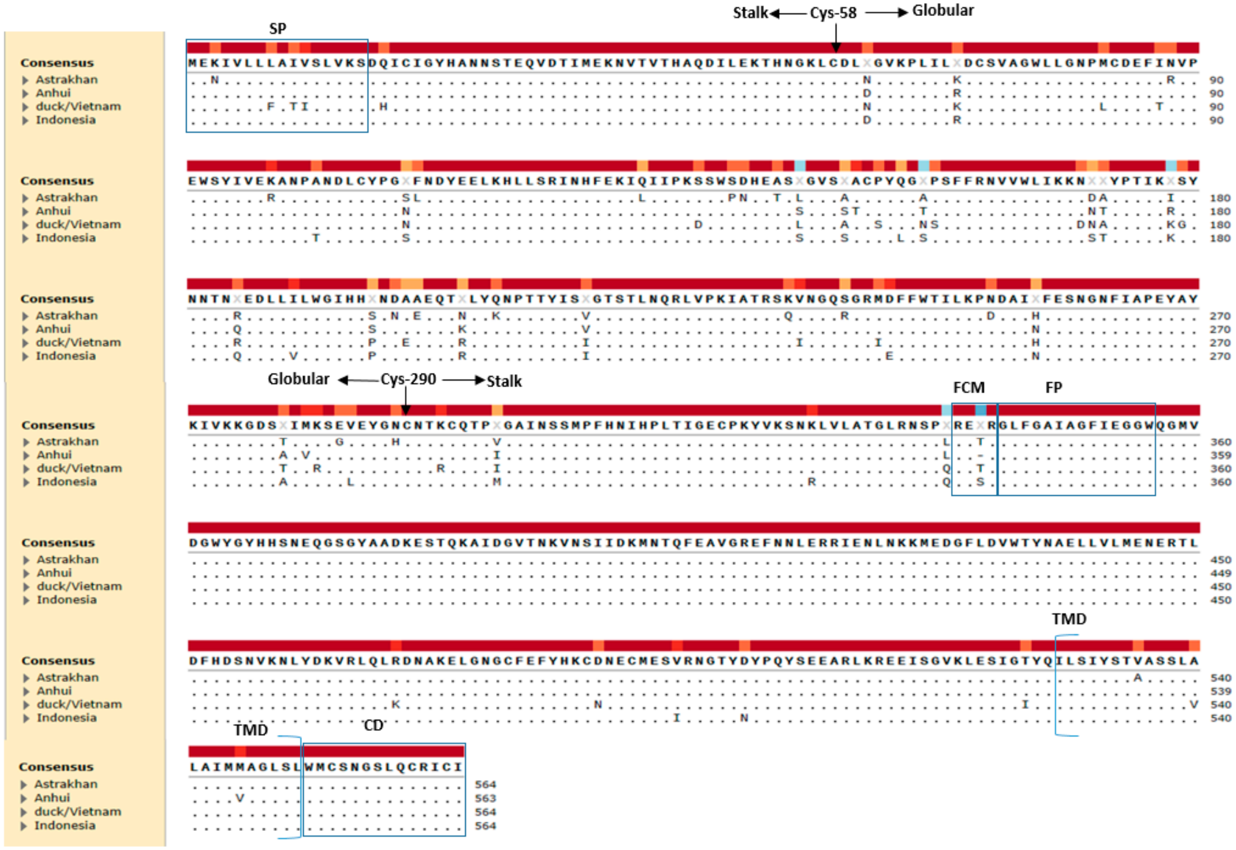

Figure S1. Multiple Sequence Alignment of the H5 amino acid sequences of the H5Nx CVVs produced at Butantan Institute (Astrakhan, Duck/Vietnam, and Anhui) and CVV Indonesia. A/Astrakhan/3212/2020(H5N8), GenBank: UJS29065; A/Anhui/1/2005(H5N1), GenBank: AXN59219; A/duck/Vietnam/NCVD-1584/2012(H5N1), GISAID EpiFlu: EPI1255841. It was also included the human licensed CVV IBCDC-RG2 A/Indonesia/5/2005(H5N1), GenBank: ABP51969, denominated as Indonesia. Some regions in the H5 sequence are highlighted: signal peptide (**SP**); furin cutting motif (**FCM**); fusion peptide (**FP**); transmembrane domain (**TMD**) and cytoplasmatic domain (**CD**).

Table S4. Different amino acids among produced CVVs (Astrakhan, Duck/Vietnam, and Anhui) and Indonesia CVV.

REGION	POSITION	IDCDC-RG71A	IBCDC-RG6	NIBRG-301	IBCDC-RG2
		Astrakhan	Anhui	Duck/Vietnam	Indonesia
		2.3.4.4b	2.3.4	2.3.2.1c	2.1.3.2
STALK	18	Q	Q	H	Q
GLOBULAR	61	N	D	N	D
	69	K	R	K	R
	82	M	M	L	M
	87	I	I	T	I
	88	R	N	N	N
	98	R	K	K	K
	102	A	A	A	T
	110	S	N	N	S
	111	L	F	F	F
	131	L	Q	Q	Q
	136	S	S	D	S
	139	P	S	S	S
	140	N	D	D	D
	143	T	A	A	A
	145	L	S	L	S
	149	A	S	A	S
	150	A	T	A	A
	152	P	P	S	P
	154	Q	Q	Q	L
	156	A	T	N	S
	157	P	P	S	P
	170	N	N	D	N
	171	D	N	N	S
	172	A	T	A	T
	178	I	R	K	K
	179	S	S	G	S
	185	R	Q	R	Q
	190	I	I	I	V
	197	S	S	P	P
	199	N	D	D	D
	200	A	A	E	A
	201	E	A	A	A
	205	N	K	R	R
	208	K	Q	Q	Q
	216	V	V	I	I
	234	Q	K	K	K
	235	V	V	I	V

Table S4. (continued)

REGION	POSITION	IDCDC-RG71A	IBCDC-RG6	NIBRG-301	IBCDC-RG2
		Astrakhan	Anhui	Duck/Vietnam	Indonesia
		2.3.4.4b	2.3.4	2.3.2.1c	2.1.3.2
GLOBULAR	239	R	S	S	S
	242	M	M	I	M
	243	D	D	D	E
	252	D	N	N	N
	256	H	N	H	N
	279	T	A	T	A
	281	M	V	M	M
	282	K	K	R	K
	284	G	E	E	E
	285	V	V	V	L
	289	H	N	N	N
STALK	293	K	K	R	K
	298	V	I	I	M
	326	K	K	K	R
	338	L	L	Q	Q
	341	T	-	T	S
	469	R	R	K	R
	487	D	D	N	D
	494	V	V	V	I
	500	D	D	D	N
	525	T	T	I	T
	535	A	V	V	V
	540	A	A	V	A
	545	M	V	M	M

2. Preparation of virus working bank

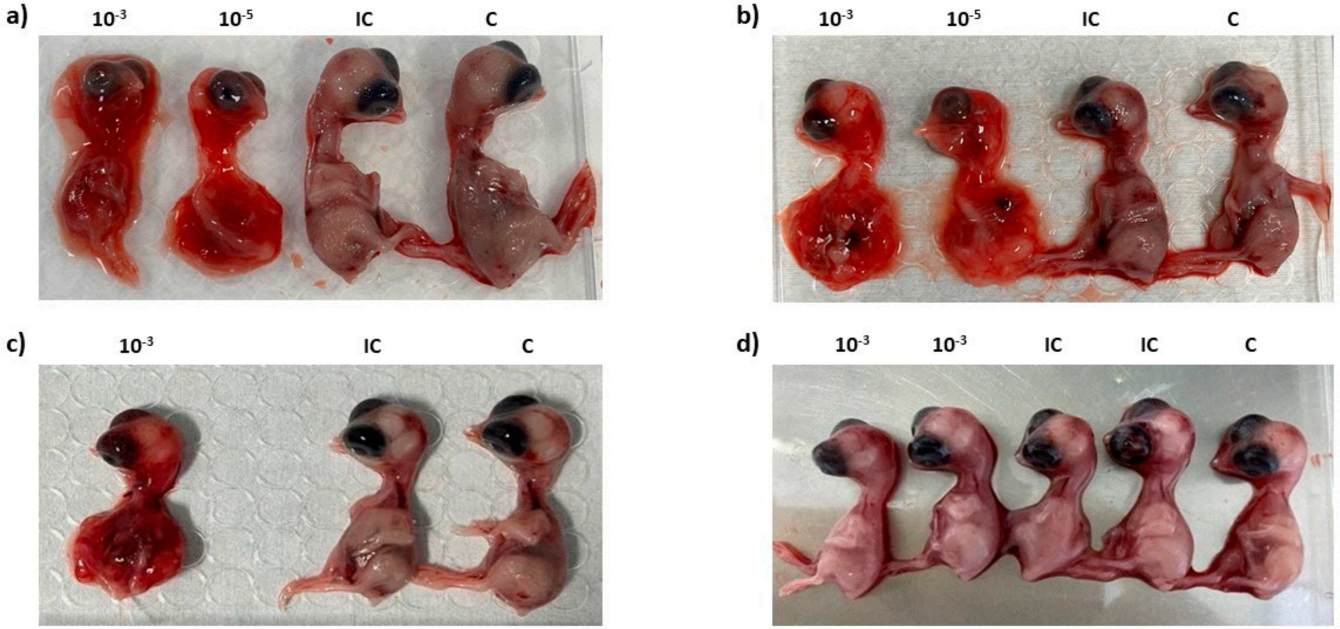

Figure S2. Embryos recovery after infection with H5Nx CVVs. Nine-day-old specific pathogen-free chicken embryonated eggs (SPF-CEE) were inoculated with 10^{-3} or 10^{-5} dilutions of the master virus seed, and incubated for 24 or 66h, respectively. Panel **(a)** IDCDC-RG71A (A/Astrakhan/3212/2020(H5N8)), **(b)** NIBRG-301 (A/duck/Vietnam/NCVD-1584/2012(H5N1)), **(c)** IDCDC-RG6 (A/Anhui/1/2005(H5N1)), and **(d)** the seasonal Influenza A/Victoria/2570/2019 (IVR-215) (H1N1) strains. Control embryos injected with PBS (IC) or without injection (C) are also shown.

3- Production of the monovalent bulks Astrakhan, Duck/Vietnam, and Anhui

Table S5. Estimated yields for H5Nx vaccines.

CVV	Batch	Bulk HA (µg/mL)	Average HA (µg/mL)	Bulk Volume (mL)	Doses/lot*	Doses/Egg*	Average Doses/Egg*
Astrakhan H5N8	2300170/00	158.01	157.43	58400	615,185.60	1.77	1.32
	2300177/00	161.55		36780	396,120.60	1.14	
	2300178/00	152.72		35620	362,659.09	1.05	
Duck/Vietnam H5N1	2301228/00	381.32	380.55	114100	2,900,574.13	8.36	9.0
	2301229/00	370.67		137630	3,401,020.81	9.81	
	2301230/00	389.66		118040	3,066,364.43	8.84	
Anhui H5N1	H5/UCG230001	276.34	266.82	64500	1,188,262.00	3.43	3.84
	H5/UCG230002	262.78		92590	1,622,053.35	4.68	
	H5/UCG230003	261.33		67830	1,181,734.26	3.41	

*One dose considered = 15 µg of HA antigen; parameters calculated based on number of theoretical doses, not considering losses and stability.

Table S6. Monovalent bulks produced for each CVV were within defined specifications.

Features	ASTRAKHAN H5N8			DUCK/VIETNAM H5N1			ANHUI H5N1			Specification
	Lot 1	Lot 2	Lot 3	Lot 1	Lot 2	Lot 3	Lot 1	Lot 2	Lot 3	
	2300170/00	2300177/00	2300178/00	2301228/00	2301229/00	2301230/00	H5/UCG230001	H5/UCG230002	H5/UCG230003	
**Virus inactivation**	Approved	Approved	Approved	Approved	Approved	Approved	Approved	Approved	Approved	Inactive virus
**Sterility (bacteria and fungi)**	Absence of bacteria and fungi growth	Absence of bacteria and fungi growth	Absence of bacteria and fungi growth	Absence of bacteria and fungi growth	Absence of bacteria and fungi growth	Absence of bacteria and fungi growth	Absence of bacteria and fungi growth	Absence of bacteria and fungi growth	Absence of bacteria and fungi growth	Absence of bacteria and fungi growth
**Endotoxin (UE/mL)**	0.242	0.108	0.1	0.1	0.5	0.5	0.18	0.1	0.307	<=100U
**pH**	7.41	7.38	7.41	7.43	7.44	7.43	7.40	7.42	7.44	6.8 – 7.6
**Visual Aspect**	Lightly opalescent liquid	Lightly opalescent liquid	Lightly opalescent liquid	Lightly opalescent liquid	Lightly opalescent liquid	Lightly opalescent liquid	Lightly opalescent liquid	Lightly opalescent liquid	Lightly Opalescent liquid	Lightly opalescent liquid
**Residual Triton X-100 (µg/mL)**	529.92	531.92	548.45	565.35	565.69	583.18	395.21	515.09	468.05	<= 920
**Residual Formaldehyde (µg/mL)**	74.98	78.72	75.73	65.47	76.91	75.08	80.94	77.19	79.07	<= 87.3
**Total Nitrogen (µg/mL)**	76.50	75.00	74.50	81.5	84.5	81.5	85.00	74.00	79.50	Informative
**Total Protein (µg/mL)**	741.38	503.25	493.13	502.75	512.87	528.12	512.15	470.14	492.20	Informative
**Ovoalbumin (ng/mL)**	348.81	592.13	768.01	30.09	18.88	19.41	42.69	48.66	27.73	Informative
**Hemagglutinin (µg/mL)**	158.01	161.55	152.72	381.32	370.67	389.66	276.34	262.78	261.33	Informative
**Hemagglutinin Identity**	Reactivity to standard antibody	Reactivity to standard antibody	Reactivity to standard antibody	Reactivity to standard antibody	Reactivity to standard antibody	Reactivity to standard antibody	Reactivity to standard antibody	Reactivity to standard antibody	Reactivity to standard antibody	Reactivity to standard antibody

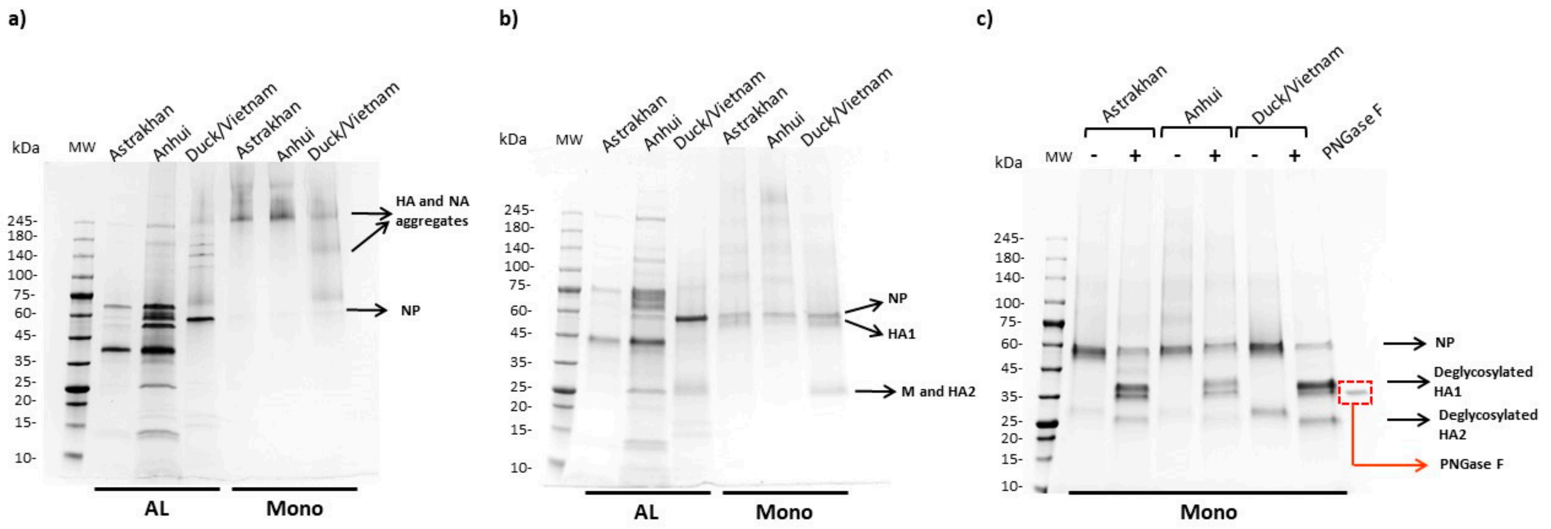

Figure S3. SDS-PAGE of allantoic liquid (AL) and final split H5Nx vaccine antigens (Mono) from A/Astrakhan/3212/2020 (H5N8), A/Anhui/1/2005 (H5N1), and A/duck/Vietnam/NCVD-1584/2012 (H5N1) CVVs. SDS-PAGE under (**a**) non-reducing conditions, (**b**) reducing conditions, and (**c**) reducing conditions with or without Peptide N glycosidase treatment ((PNGase F +) or (PNGase F -), respectively). Mono: allantoic liquid after clarification by continuous centrifugation and concentration by a tangential filtration step, fractionation by two zonal centrifugations on saccharose cushion, selected fraction were properly diluted and treated with Triton-X100, clarified and diafiltrated by tangential filtration, diluted and inactivated with formaldehyde and sterilized by 0.22 µm filtration (see Material and Methods section). **MW**- molecular mass protein standards. **HA1** and **HA2**- Hemagglutinin fragments 1 and 2, respectively. **NA**- Neuraminidase. **NP**- Nucleoprotein. **M**- Matrix protein.

4- Tolerability and safety of vaccine formulations by weight gain

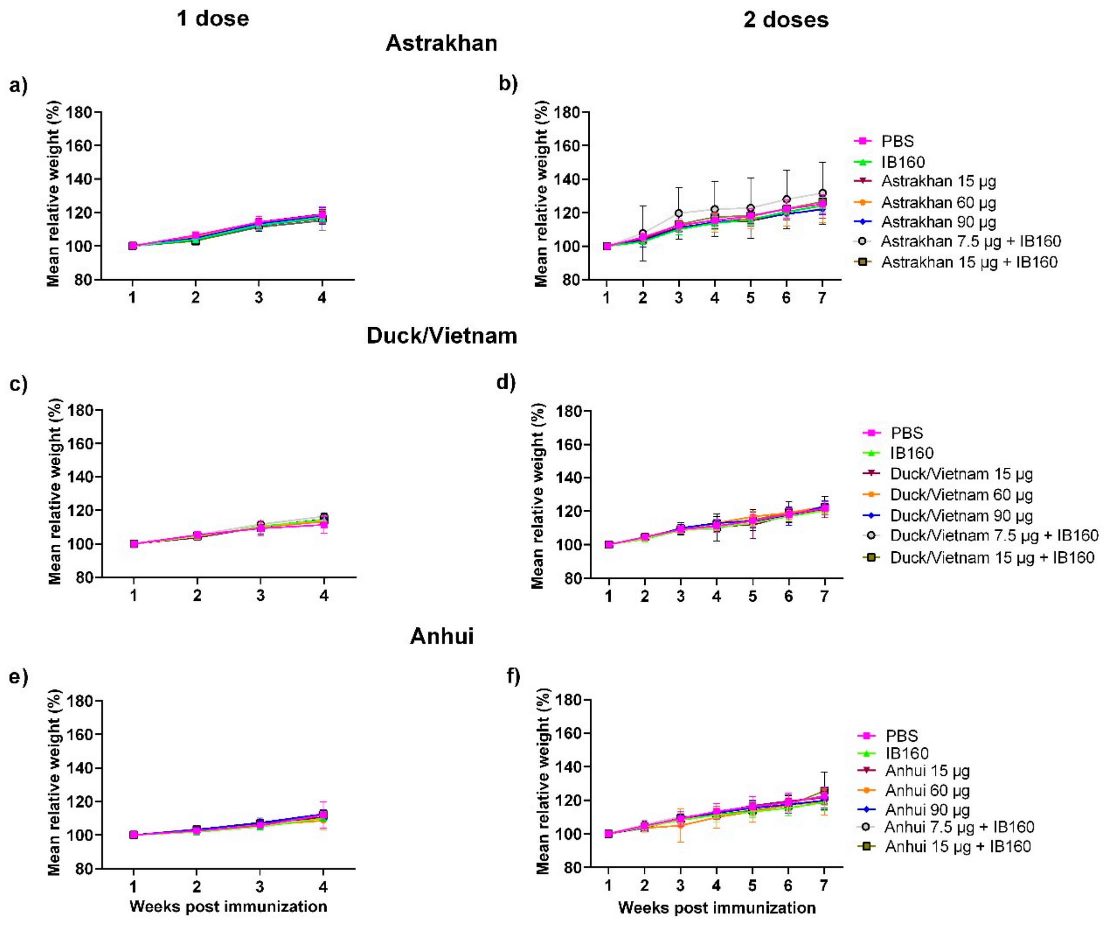

Figure S4. Weight loss/gain curves of rats immunized with the H5Nx vaccine formulations. The relative weight (weekly weight / initial weight) is expressed in percentage and was analyzed over the experimental period, five or eight weeks according to immunization protocol. The graphics illustrate the arithmetic mean of relative weight of the groups receiving one (N=5) or two (N=6) doses of the Astrakhan (H5N8) (panels **a** and **b**), the Duck/Vietnam (H5N1) (panels **c** and **d**), or the Anhui (H5N1) (panels **e** and **f**) vaccine formulations.

5- CVVs belonging to clade 2.3.4.4b

We also analyzed the similarity of the amino acid H5 sequence of CVVs belonging to clade 2.3.4.4b (IDCDC-RG71A and IDCDC-RG78A) with the hemagglutinin of a bird isolate reported in Brazil and human isolates reported in the United States (Table S7). They are EPI_ISL_18130597 (A/Thalasseus_maximus/Brazil-ES/23ES1A0008/2023 (H5N1), genotype B3.2, H5 GISAID Epiflu: EPI2701038); EPI_ISL_19294964 (A/Colorado/139/2024 (H5N1), genotype B3.13, H5 GISAID Epiflu: EPI3467521); and EPI_ISL_19634827 (A/Louisiana/12/2024 (H5N1), genotype D1.1, H5 GISAID Epiflu: EPI3741715) isolates. For CVVs, we tagged the H5 sequence of the antigenic prototype: GenBank: WHA12325.1 (IDCDC-RG78A (A/American wigeon/South Carolina/22- 000345-001/2021-like (H5N1)) and GISAID Epiflu: EPI1846961 (IDCDC-RG71A (A/Astrakhan/3212/2020(H5N8)). Multiple sequence alignment among all the sequences is shown in Figure S5. Different amino acids among them are listed in Table S8, as well as the position and domain in the protein. Comparing the five sequences, they were found twelve positions with different amino acids: six in the globular domain and six in the stalk region.

Table S7. Identity of H5 hemagglutinin among CVVs belonging to clade 2.3.4.4b and American H5N1 isolates.

	Identity of H5 globular domain[a] (%)[b]				
**H5 access number**	**EPI2701038**	**EPI3467521**	**EPI3741715**	**IDCDC-RG78A**	**IDCDC-RG71A**
**EPI2701038 (Brazil)**	100	99.14	98.28	99.14	98.28
**EPI3467521 (Colorado)**	99.14	100	98.28	99.14	98.28
**EPI3741715 (Louisiana)**	98.28	98.28	100	99.14	100
**WHA12325.1 (IDCDC-RG78A)**	99.14	99.14	99.14	100	99.14
**EPI1846961(IDCDC-RG71A)**	98.28	98.28	100	99.14	100

[a]globular domain delimited by the conserved Cys 58 and 290 residues (H5 numbering)
[b]% of identity among isolates (yellow), isolates and CVVs (green), and CVVs (blue)

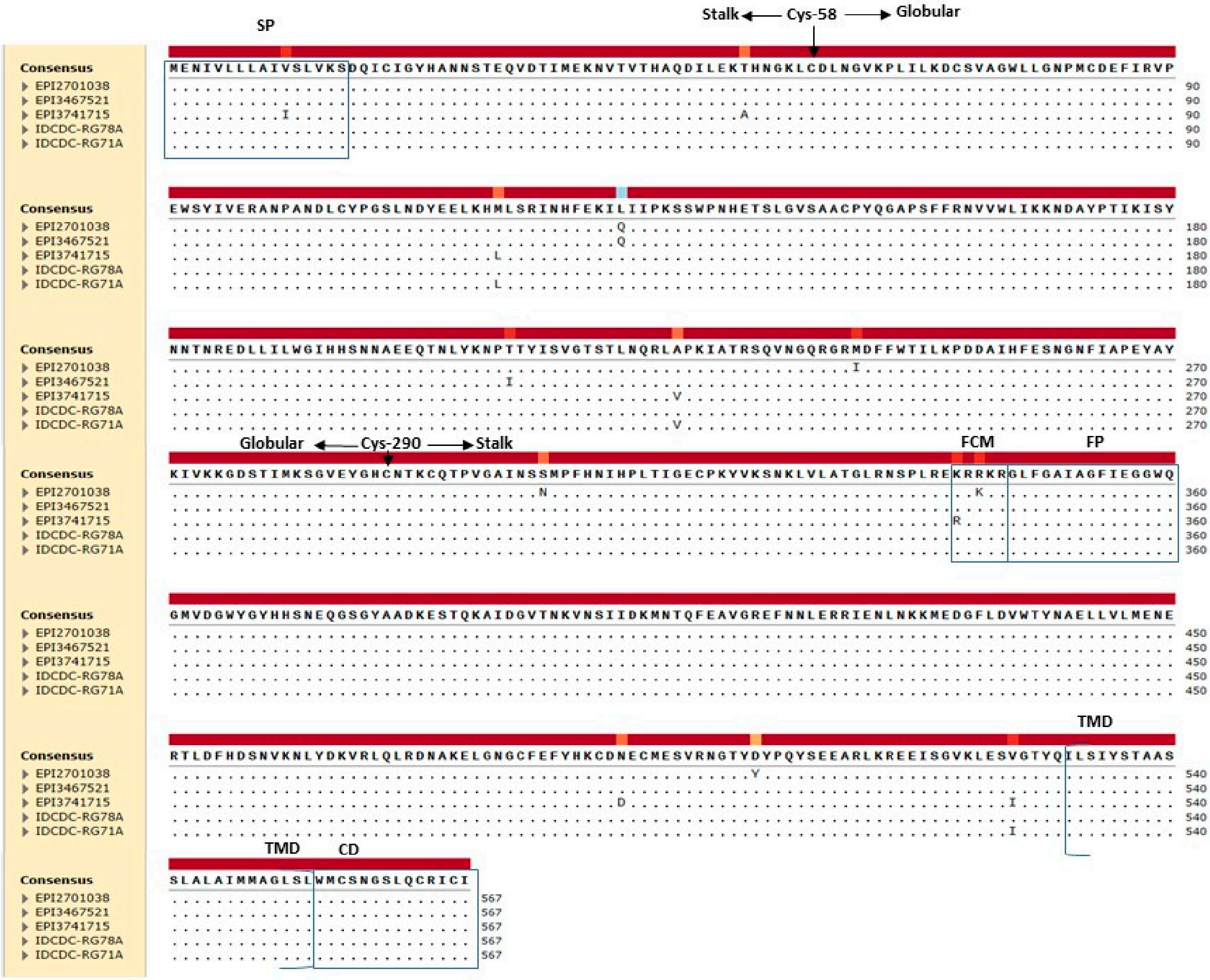

Figure S5. Multiple Sequence Alignment of the H5 amino acid sequences among 2.3.4.4b H5Nx CVVs and H5N1 isolates. Antigenic prototype of CVVs: IDCDC-RG71A (A/Astrakhan/3212/2020(H5N8), GISAID Epiflu: EPI1846961) and IDCDC-RG78A (A/American wigeon/South Carolina/22- 000345-001/2021-like (H5N1), GenBank: WHA12325.1). Bird isolate: EPI_ISL_18130597 (A/Thalasseus_maximus/Brazil-ES/23ES1A0008/2023 (H5N1), genotype B3.2, GISAID Epiflu: EPI2701038), and human isolates: EPI_ISL_19294964 (A/Colorado/139/2024 (H5N1), genotype B3.13, GISAID Epiflu: EPI3467521) and EPI_ISL_19634827 (A/Louisiana/12/2024 (H5N1), genotype D1.1, GISAID Epiflu: EPI3741715). Some regions in the H5 sequence are highlighted: signal peptide (**SP**); furin cutting motif (**FCM**); fusion peptide (**FP**); transmembrane domain (**TMD**) and cytoplasmatic domain (**CD**).

Table S8. Different amino acids among H5 sequences of 2.3.4.4b CVVs and H5N1 isolates.

REGION	POSITION	H5 sequences of isolates[a]			H5 sequences of CVVs[b]	
		Brazil EPI2701038[c]	Colorado EPI3467521[d]	Louisiana EPI3741715[d]	IDCDC-RG78A WHA12325.1	IDCDC-RG71A EPI1846961
GLOBULAR	75	T	T	A	T	T
	120	M	M	L	M	L
	131	Q	Q	L	L	L
	211	T	I	T	T	T
	226	A	A	V	A	V
	242	I	M	M	M	M
STALK	304	N	S	S	S	S
	341	K	K	R	K	K
	343	K	R	R	R	R
	492	N	N	D	N	N
	502	Y	D	D	D	D
	526	V	V	I	V	I

[a]belonging to clade 2.3.4.4b

[b]H5 amino acid sequences of the antigenic prototypes of the CVVs IDCDC-RG71A (A/Astrakhan/3212/2020(H5N8) and IDCDC-RG78A (A/American wigeon/South Carolina/22- 000345-001/2021-like (H5N1).

[c]bird isolate: GISAID Epiflu: EPI_ISL_18130597 (A/Thalasseus_maximus/Brazil-ES/23ES1A0008/2023 (H5N1), genotype B3.2, H5: EPI2701038)

[d]human isolates: GISAID Epiflu: EPI_ISL_19294964 (A/Colorado/139/2024 (H5N1), genotype B3.13, H5: EPI3467521) and EPI_ISL_19634827 (A/Louisiana/12/2024 (H5N1), genotype D1.1, H5: EPI3741715, deceased).
